# Choosing Optimal Seed Nodes in Competitive Contagion

**DOI:** 10.3389/fdata.2019.00016

**Published:** 2019-06-20

**Authors:** Prem Kumar, Puneet Verma, Anurag Singh, Hocine Cherifi

**Affiliations:** ^1^Department of Computer Science and Engineering, National Institute of Technology Delhi, New Delhi, India; ^2^LIB EA 7534, University of Burgundy, Dijon, France

**Keywords:** competitive contagion, complex networks, game theory, seed nodes, competitive marketing, centrality measures

## Abstract

In recent years there has been a growing interest in simulating competitive markets to find out the efficient ways to advertise a product or spread an ideology. Along this line, we consider a binary competitive contagion process where two infections, A and B, interact with each other and diffuse simultaneously in a network. We investigate which is the best centrality measure to find out the seed nodes a company should adopt in the presence of rivals so that it can maximize its influence. These nodes can be used as the initial spreaders or advertisers by firms when two firms compete with each other. Each node is assigned a price tag to become an initial advertiser which varies according to their importance in the network. Considering their fixed budgets, they initially determine the payoff of their products and the number of their initial seeds in the network. Under this setting, we study the question of whether to choose a small number of influential nodes or a larger number of less influential nodes.

## 1. Introduction

Contagion in general life means the communication of disease from one person or organism to another by close contact. This definition can be extended by replacing the disease with a product or an ideology. Competitive Contagion is a type of contagion which deals with conflict and race of multiple firms who want to influence or infect more people than others. There are a lot of situations that can be described in such a way, for example: Two political parties trying to influence the citizens by giving incentives to some influential people in the country and directing them to advertise their ideology, Two mobile phone manufactures competing to advertise their mobile phones of same segment by hiring celebrities or tech reviewers and giving them incentives. So, it is important to simulate such an environment and provide algorithms and properties for optimal seed selection for the competitive contagion process. While doing such competition this work can be used by firms to select the initial spreaders or advertisers by analyzing their network topological properties.

Diffusion on networks is a fundamental process which involves spreading of an ideology (or infection) in a population, e.g., epidemic disease contagion, spread of innovation by word-of-mouth. Considering a network diffusion model, the influence maximization problem consists of finding a set of initial seed nodes so that the expected size of the resulting cascade is maximized. Supposing that there is a limit k on the number of nodes to target (e.g., due to advertising budgets), the goal is to efficiently find an appropriate set of k nodes with which to “seed” a diffusion process. Classical works by Kempe et al. (2003, 2005), on this subject are competitive unaware. They focused on designing models of spreading of a single influence (or idea) and algorithms to find out the optimal seed nodes for maximal adoption of a product of a single firm only. However in real life scenarios, several firms compete in the same market and multiple infections can occur simultaneously in the same network. This has led to the increase in effort toward finding seeds for more realistic settings. Recent works by Bharathi et al. (2007) and Goyal et al. (2014) focused on the modeling the competitive contagion of multiple firms using game theory. They proposed an algorithm to select the seed nodes in a network and discussed the Nash equilibrium when multiple firms compete against each other. Despite the considerable progress made toward finding the seeds in the social network in competitive settings, some very basic questions remain unanswered. Indeed, one approach used to make the influence maximization is to reduce the problem into the ranking of the nodes according to the centrality measures. In other words, the seed nodes are selected by mentioning its ranks using various centrality measure. This raises an important question as to which of the centrality measures should firms use to rank the nodes while selecting them as seed nodes. To answer this question, we compare various centrality methods for finding the rank of nodes. In order to do so, for all the centralities under investigation, we assign a seed strategy based on a centrality to one firm and another to the other one and we compare their spreading efficiency. We also address the question whether a firm should select a small number of highly influential nodes or a larger number of less influential nodes. To answer this question we assign each node a price tag using the best centrality measure found during our analysis and give a fixed and same amount of budget to both firms. Then one firm stakes his funds in buying large number of cheap seed nodes and other in buying small number of expensive seed nodes and then we compare their influence or number of nodes infected when stability is achieved.

The rest of paper is organized as follows: In section 2 basic terminologies and definitions mentioned in the paper are recalled. In section 3, the diffusion model is presented. Section 4 deals with comparing the efficiency of classical centrality measures in the competitive contagion in order to choose the seed nodes. Section 5 compares the strategies of using few highly influential seeds rather than a higher number of less influential seeds with the same budget. Section 6 concludes the paper.

## 2. Background

We can classify the contagion processes (Dassios and Zhao, 2011) into three categories based on the dependency of one disease A to another disease B:

1. Competitive: This type of process occurs only if there are multiple diseases (or information about products or ideologies) to propagate. Here, if a node is already infected by a disease *A* it resists the infection by another disease *B*, e.g., diffusion of ideology of two political parties.
2. Cooperative: It is just the opposite of competitive contagion. Here, if a node is already infected by a disease *A*, and another infection *B* is trying to infect it, disease *A* helps disease *B* to infect the node. e.g., : Diffusion of two diseases (Tuberculosis and common flu).
3. Independent: As the name suggests in this type of contagion no infection (or information about product) interacts with each other and are independent.

We need to calculate the importance of a node in the network to assign its price. Higher rank nodes will be considered costlier in comparison with lower rank nodes. Centrality is a measure for calculating the importance of a node based on its topological properties in the network. There are numerous centrality measures based on various topological properties of nodes which are used in order to assign a score of importance to every node (Gupta et al., 2015, 2016). In this work we restrain our attention to the most influential measures. Their definitions are given below:

1. Degree Centrality: It considers that the node centrality is linked to the size of its neighborhood. It is simply the number of nodes at a distance of one edge.
2. Closeness Centrality: It considers nodes having smaller distance with all other nodes to be more central.
  $$Closeness(v)=\frac{1}{\sum_{i\neq v}d_{vi}}$$
  - where *d*_*vi*_ is distance between node v to i.
3. Betweenness Centrality: It works on the concept that the more often a node acts as a bridge along the shortest path between any two nodes, the more central it is.
  $$Betweeness\left(v\right)=\underset{s\neq v\neq t\in V}{\sum }\frac{\sigma_{st}\left(v\right)}{\sigma_{st}}$$
  - where σ_*st*_ is total number of shortest paths from node s to node t and σ_*st*_(*v*) is the number of those paths that pass through v.
4. EigenVector Centrality: It works on the concept that connections to high-scoring nodes contribute more to the score of the node in question than equal connections to low-scoring nodes. For a given graph *G*: = (*V, E*), Let *A* be the adjacency matrix.
  Ax=λx
  - where λ is the eigenvalue and x is the resulting eigenvector which contains the centrality measure of *ith* node at *ith* row.
  There will be multiple eigenvalues λ for which non-zero solution exists. However, (by the Perron, 1907; Frobenius, 1912 theorem) only the greatest eigenvalue results in desired centrality measure.
5. Page Rank Centrality (Page et al., 1999): It is a variant of the EigenVector Centrality. It works on the assumption that more important nodes are likely to receive more links from other nodes.

$$PR\left(u\right)\propto \underset{v\in B_{u}}{\sum }\frac{PR\left(v\right)}{L\left(v\right)}$$

i.e., the PageRank value for a page *u* is dependent on the PageRank values for each page *v* contained in the set *B*_*u*_ (the set containing all pages linking to page *u*), divided by the number *L*(*v*) of links from page *v*. The algorithm involves a damping factor for the calculation of the pagerank.

## 3. Diffusion Process

We study a competitive process of adoption of multiple products made by multiple firms who use their respective monetary resources for advertisement of their product to the consumers located in a network. Each firm has a fixed budget to advertise their products to the users in a social network. Therefore, each firm needs to optimally choose a set of seed nodes using the assigned budget for maximum adoption of their product. We use the generic game theoretic model (Osborne and Rubinstein, 1994) for the study of competition between firms. In view of game theoretic scenario in competitive market, we propose a diffusion algorithm for the spreading of any information about a product.

**The proposed game theoretic model may be represented as:**

**Players:** The firms (A and B).
**Actions:** Each firm's set of actions is to choose the initial seed nodes or their advertisers.
**Preferences:** Each firm's preference is to maximize the adoption of their product in the network or to infect the maximum number of nodes possible.

**Figure 1:**
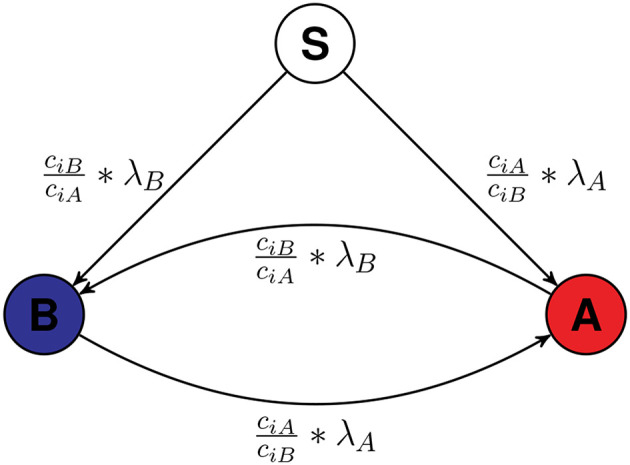
The model of spreading. The transparent node labeled as S is a Susceptible node which is not yet influenced by either firms. Initially all the nodes except seed nodes are Susceptible and they can be influenced by A or B. Once infected by either A or B, they will stay infected. As the scenario is competitive, a node infected by A can be converted to a node infected by B and vice versa, if the required condition is satisfied. The value along the arrow is the probability of infection. The node is assumed as the *i*th labeled node.

Multiple firms may try to spread information about their products in the underlying social network. Here, in this work two firms are considered for spreading information about their two products, respectively. As the only action provided to firms is to choose the seed nodes at the beginning, so the result of the entire game depends on the strategy to choose the set of starting spreader (or seed) nodes. Each of the firms (*A* and *B*) comes into the open market to advertise their product with limited budget *C*_*A*_ and *C*_*B*_. They have their node ranking algorithm using which they rank the nodes present in the network and then select the nodes whose price is less than their remaining budget, starting from highest rank (numerically lowest: Rank 1) till the funds remained are not enough to hire any node. The proposed diffusion Algorithm 1 used to simulate the dynamics is an extension of a previous work on simulating epidemic and rumor spreading (Kumar et al. 2018) in which we proposed a simple cascade algorithm for diffusion, discussed various characteristics of epidemic and rumor spreading and the relation among various attributes of epidemic and rumor spreading. This algorithm is a cascade based algorithm in which at each timestamp all the infected nodes try to transmit their disease (or ideology) to their direct connections and the probability that infection will transmit depends upon λ_*A*_, λ_*B*_, *c*_*iA*_, and *c*_*iB*_. Figure 1 shows the conversion model of nodes based on Algorithm 1.

λ_*A*_ and λ_*B*_ are the infection rates of infection A and infection B which are constant. *c*_*iA*_ and *c*_*iB*_ are the competitive measures of node *i*. It is 1 at time *t* = 0 for every node and changes when the node is infected by any infection. When *ith* node is infected by A, *c*_*iA*_ gets multiplied by α_*A*_ and if *jth* node is infected by B, *c*_*jB*_ gets multiplied by α_*B*_. α_*A*_ and α_*B*_ are the competitive constants, larger the α_*X*_ more is the resistance of a node infected by X for another infection.

The population is divided into two compartments: Susceptible and Infected and infected is further divided into two compartments: Infected by A and Infected by B. Let N be the total population, *S* be the number of Susceptible nodes, *X*_*A*_ and *X*_*B*_ be the number of seed nodes of *A* and *B*, *I*_*A*_ and *I*_*B*_ be the number of nodes infected by *A* and *B* respectively. At the start of simulation *I*_*A*_ = *X*_*A*_ and *I*_*B*_ = *X*_*B*_.

$$\begin{array}{r} The\;law\;of\;conservation\;will\;be:N=S+I_{A}+I_{B}\; \end{array}$$

(1) $$\begin{array}{r} where{\;X}_{A}\subset I_{A}\;\&\;X_{B}\subset I_{B} \end{array}$$

## 4. Finding the Optimal Centrality Measure

The agents present in the network take a fixed amount to advertise or spread the product of the firms and that value is decided in accordance to centrality value of the agents. To find out which centrality measure is more effective for finding the most influential nodes in a competitive contagion scenario, we compare the following five centrality measures: Page Rank, Degree, Betweenness, Closeness, EigenVector. To do so, we consider each method as a node ranking algorithm of a firm trying to advertise its product. Therefore, there are total $\left(\begin{array}{c} 5\\ 2 \end{array}\right)$ matches (*Match of every centrality against every other centrality*. Each firm ranks the top 10 nodes according to their node ranking algorithm and put them in their seed nodes set. As there are cases in which both competing centralities have common nodes in their top 10 list, we assign only unique nodes to each. An example of this distribution is given in Table S2. Table S2 contains the list of seed nodes for competitions of various centrality measures. After making the set of seeds for each match, we run the simulation for dynamics of infection using the Algorithm 1.

**Table:** Algorithm 1 Diffusion algorithm.

1:	*G*: Population (Graph).
2:	*S*_*A*_, *S*_*B*_: The set of seed nodes of firms *A* and *B* respectively.
3:	λ_*A*_, λ_*B*_: Probability of spreading information about the product of *A* and *B* firms respectively.
4:	*c*_*iA*_, *c*_*iB*_: competitive measures for Firms *A* and *B* respectively.
5:	α_*A*_, α_*B*_: competitive constant for respectively of *A* and *B*.
6:	For each node, i other than the initial seeds *S*_*A*_∪*S*_*B*_:
7:	**procedure** (*G*, *S*_*A*_, *S*_*B*_, λ_*A*_, λ_*B*_, *c*_*iA*_, *c*_*iB*_, α_*A*_, α_*B*_)
8:	Count the number of neighbors infected by A (*n*_*A*_) and B (*n*_*B*_) and respectively
9:	*x* ← *n*_*A*_ * λ_*A*_ * *c*_*iA*_/*c*_*iB*_
10:	*y* ← *n*_*B*_ * λ_*B*_ * *c*_*iB*_/*c*_*iA*_
11:	**if** *x* > *y* **then**
12:	Generate a random number (*r*_0_) between 1 and 100.
13:	**if** *r*_0_ < λ_A_*c_*iA*_/*c*_*iB*_ **then**
14:	*node*_*i*_ gets infected by A
15:	*c*_*iA*_ ← *c*_iA_*α_*A*_
16:	**end if**
17:	**else**
18:	generate another random number (*r*_1_) between 1 and 100.
19:	**if** *r*_1_ < λ_B_*c_*iB*_/*c*_*iA*_ **then**
20:	*node*_*i*_ gets infected by B
21:	*c*_*iB*_ ← *c*_iB_*α_*B*_
22:	**end if**
23:	**end if**
24:	**if** *x* < *y* **then**
25:	Generate a random number (*r*_2_) between 1 and 100.
26:	**if** *r*_2_ < λ_B_*c_*iB*_/*c*_*iA*_ **then**
27:	*node*_*i*_ gets infected by B
28:	*c*_*iB*_ ← *c*_iB_*α_*B*_
29:	**end if**
30:	**else**
31:	generate another random number (*r*_3_) between 1 and 100.
32:	**if** *r*_3_ < λ_A_*c_*iA*_/*c*_*iB*_ **then**
33:	*node*_*i*_ gets infected by A
34:	*c*_*iA*_ ← *c*_iA_*α_*A*_
35:	**end if**
36:	**end if**
37:	**end procedure**

## 5. Choosing the Type and Number of Seeds

A general confusion among the firms is whether to choose small number of highly influential advertisers or large number of less or average influential advertisers. To solve this problem, we simulate a competition between a large group of less (or average) influential nodes and a small group of highly influential nodes both needing nearly same amount of budget.

For ranking the nodes while simulating the competition between group of small number of highly influential nodes and group of large number of less influential nodes we will use the most optimal centrality method found during the simulation discussed in section 4. We select the two sets such that both of them cost nearly same.

(2) $$\begin{array}{r} Cost\propto Centrality\;score \end{array}$$

To investigate the *high-less* (highly influential nodes in small numbers) vs. *low-more* (low influential nodes in large number) competition. We took a set of less influential nodes mostly from different clusters in the *low-more* set and most influential nodes in the *high-less* set such that the cost of both is same.

## 6. Results and Analysis

We use three empirical network data-sets to perform the experiments [the wikipedia vote network which is available on: SNAP Stanford^1^, the chess interaction network which is available on KONECT^2^], and the human contact network available on KONECT^3^. We have used the Wikipedia Vote Network as our primary data-set and others for verification. The results for networks other than chess interaction is added in the Supplementary Material. Details of the basic topological properties of all networks is given below in Table 1.

**Table 1:** Properties of data-sets used.

**Network**	**Nodes**	**Edges**	**Av. Clustering Coe**.	**Diameter**
Wikipedia vote	7,115	103,689	0.1409	7
Chess interaction	7,301	65,053	0.126	13
Human interaction	410	2,765	0.436	9

### 6.1. Finding the Optimal Centrality Measure

The simulation proposed in section 4 is run on a fixed rate of spreading (λ_*A*_ = λ_*B*_ = 0.6) and fixed competitive constant (α_*A*_ = α_*B*_ = 1.1) for both firms for 100 timestamps and for each timestamp, average of 50 iterations are considered. Output of the simulation is the ratio of nodes infected by each firm after each timestamp. Table 2 shows the results of matches among various centrality methods when simulated with various data sets. The individual curves for the matches (Fraction of nodes infected by each firm vs. time) is provided in Supplementary Material.

**Table 2:** Results of various competitions on various network datasets.

**Player 1**	**Player 2**	**Winner-Wiki**	**Winner-Chess**	**Winner-Human**
Pagerank	EigenVector	**EigenVector**	**Eigenvector**	**Pagerank**
Closeness	EigenVector	**Closeness**	**Closeness**	**Closeness**
Betweenness	EigenVector	**EigenVector**	**Eigenvector**	**Betweenness**
Degree	EigenVector	**EigenVector**	**Degree**	**Degree**
Pagerank	Betweenness	**Betweenness**	**Pagerank**	**Pagerank**
Betweenness	Closeness	**Closeness**	**Closesness**	**Tie**
Degree	Betweenness	**Degree**	**Degree**	**Degree**
Degree	Closeness	**Closeness**	**Degree**	**Degree**
Pagerank	Degree	**Degree**	**Degree**	**Pagerank**
Pagerank	Closeness	**Closeness**	**Closeness**	**Pagerank**

*Bold column values show the winners of respective matches*.

The simulation results (Table 2) shows that no centrality performs best in competitive setting of contagion and it is data dependent.

### 6.2. Choosing the Type and Number of Seeds

As proposed in section 5, we simulated the competition between two firms, one having higher number of less influential node and one having small number of highly influential node using all the three datasets. As we have seen that none of centrality is best for all datasets but it is data dependent so, we will use the centrality method which performed best for that particular dataset. So for Wikipedia Vote Network it will be Closeness, for Chess Interaction it will be Degree, and for Human Interaction network it will be Pagerank.

**Figure 2:**
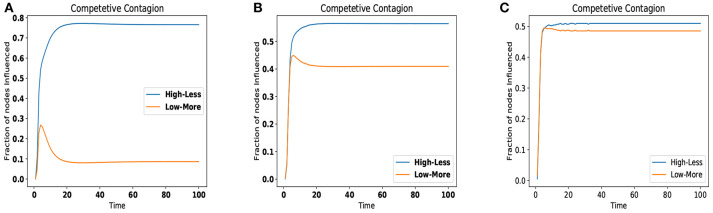
Competition of High-Less vs. Low-More for various datasets. **(A)** Wikipedia Vote Network. **(B)** Chess interaction network. **(C)** Human interaction network.

As the Figure 2 depicts for all three datasets, the number of infected (or influenced) nodes remains same for both the sets up to few timestamps, but after that *High-less* set takes over then, stabilization is achieved. Overall winner is *High-less* (less number of highly influential nodes) if we use the better performing centrality measure as per datasets to assign the costs of nodes. For further verification we used a synthetic dataset, but in the case of synthetic dataset all the centralities demand the nearly the same nodes at each rank to it is not possible to allocate the nodes to any centrality and simulate the competition of centralities.

## 7. Conclusion

In this paper, we investigate empirically two linked issue in a competitive contagion setting. First, we simulate a set of competitions between strategies based on ranking according to various centrality measures. The goal is to choose the best centrality method to rank the nodes for initial adoption with a motive to maximize the adoption of the product or ideology. Results show that no centrality is universally best but it depends on the network properties of the network dataset used. The second part deals with solving the general dilemma of whether to choose group of small number of highly influential nodes or a group of large number of less influential nodes. We conclude that it is better to select a small number of highly influential nodes than a higher number of less influential nodes. We can extend this work by taking variable rate of spreading, cooperativity and competitive constant of the diffusion model. Future works could also be done by considering more sophisticated alternative network properties for selecting the seed nodes.

## Data Availability

The datasets analyzed for this study can be found http://snap.stanford.edu/data/ego-Facebook.html.

## Author Contributions

PK and PV discussed the idea with AS. PK, PV, AS, and HC designed the methodology for the experiments. PK did the simulation and data pre-processing. PV did the data representation and image editing. AS and HC guided and helped with bugs and theoretical understanding. All authors wrote the article.

### Conflict of Interest Statement

The authors declare that the research was conducted in the absence of any commercial or financial relationships that could be construed as a potential conflict of interest.

## Footnotes

^1^(accessed February 3, 2019). *SNAP: Network datasets: Social circles*.

^2^(accessed February 25, 2019). *KONECT Networks*.

^3^(accessed May 13, 2019). *KONECT Networks*.
