# Supplementary material for: Choosing Optimal Seed Nodes in Competitive Contagion
Source: Front Big Data. 2019 Jun 20;2:16. doi: 10.3389/fdata.2019.00016 (PMC7931913; doi:10.3389/fdata.2019.00016)
Supplement: Supplementary file 1 [file Data_Sheet_1.PDF]

# Supplementary Material

## 1 SUPPLEMENTARY DATA FOR WIKI VOTE NETWORK

**Table S1.** This set of tables show the seed nodes of each centrality methods for Wikipedia Vote Network. Seed nodes here are the unique nodes selected from the top 10 nodes of each centrality measures. Blue coloured cells show the nodes which are not unique and can't be considered as a seed node if the distribution of seed nodes is unbiased. For example: In the first set. e.g. In the first part of table EigenVector demands 766 at rank 2 but 766 has already been allotted to Pagerank at rank 1, so no one is given the node correspondingly ranked 2 by them; In the sixth part of table both Betweenness and Closeness demands 2565, so both of them are not given that node as the game is unbiased; In the seventh part of table Degree demands 11 at rank 3 but 11 has already been allotted to Betweenness at rank 2, so no one is given their corresponding node at rank (11 to Degree and 457 to Betweenness), further at rank 5 Degree demands 457 which was denied to Betweenness at rank 3, so both of them are not given the node ranked 5 by them

| | | | | | | | | | | |
|---|---|---|---|---|---|---|---|---|---|---|
| **Pagerank** | 766 | 457 | 4037 | 1549 | 1166 | 2688 | 15 | 1374 | 2237 | 5524 |
| **EigenVector** | 2565 | 766 | 1549 | 1166 | 2688 | 457 | 3352 | 11 | 1151 | 1374 |

| | | | | | | | | | | |
|---|---|---|---|---|---|---|---|---|---|---|
| **Closeness** | 2565 | 766 | 457 | 1549 | 1166 | 1374 | 11 | 1151 | 2688 | 2485 |
| **EigenVector** | 2565 | 766 | 1549 | 1166 | 2688 | 457 | 3352 | 11 | 1151 | 1374 |

| | | | | | | | | | | |
|---|---|---|---|---|---|---|---|---|---|---|
| **Betweenness** | 2565 | 11 | 457 | 4037 | 1549 | 766 | 1166 | 15 | 1374 | 2237 |
| **EigenVector** | 2565 | 766 | 1549 | 1166 | 2688 | 457 | 3352 | 11 | 1151 | 1374 |

| | | | | | | | | | | |
|---|---|---|---|---|---|---|---|---|---|---|
| **Degree** | 2565 | 766 | 11 | 1549 | 457 | 1166 | 2688 | 1374 | 1151 | 5524 |
| **EigenVector** | 2565 | 766 | 1549 | 1166 | 2688 | 457 | 3352 | 11 | 1151 | 1374 |

| | | | | | | | | | | |
|---|---|---|---|---|---|---|---|---|---|---|
| **Pagerank** | 766 | 457 | 4037 | 1549 | 1166 | 2688 | 15 | 1374 | 2237 | 5524 |
| **Betweenness** | 2565 | 11 | 457 | 4037 | 1549 | 766 | 1166 | 15 | 1374 | 2237 |

| | | | | | | | | | | |
|---|---|---|---|---|---|---|---|---|---|---|
| **Betweenness** | 2565 | 11 | 457 | 4037 | 1549 | 766 | 1166 | 15 | 1374 | 2237 |
| **Closeness** | 2565 | 766 | 457 | 1549 | 1166 | 1374 | 11 | 1151 | 2688 | 2485 |

| | | | | | | | | | | |
|---|---|---|---|---|---|---|---|---|---|---|
| **Degree** | 2565 | 766 | 11 | 1549 | 457 | 1166 | 2688 | 1374 | 1151 | 5524 |
| **Betweenness** | 2565 | 11 | 457 | 4037 | 1549 | 766 | 1166 | 15 | 1374 | 2237 |

| | | | | | | | | | | |
|---|---|---|---|---|---|---|---|---|---|---|
| **Degree** | 2565 | 766 | 11 | 1549 | 457 | 1166 | 2688 | 1374 | 1151 | 5524 |
| **Closeness** | 2565 | 766 | 457 | 1549 | 1166 | 1374 | 11 | 1151 | 2688 | 2485 |

| | | | | | | | | | | |
|---|---|---|---|---|---|---|---|---|---|---|
| **Pagerank** | 766 | 457 | 4037 | 1549 | 1166 | 2688 | 15 | 1374 | 2237 | 5524 |
| **Degree** | 2565 | 766 | 11 | 1549 | 457 | 1166 | 2688 | 1374 | 1151 | 5524 |

| | | | | | | | | | | |
|---|---|---|---|---|---|---|---|---|---|---|
| **Pagerank** | 766 | 457 | 4037 | 1549 | 1166 | 2688 | 15 | 1374 | 2237 | 5524 |
| **Closeness** | 2565 | 766 | 457 | 1549 | 1166 | 1374 | 11 | 1151 | 2688 | 2485 |

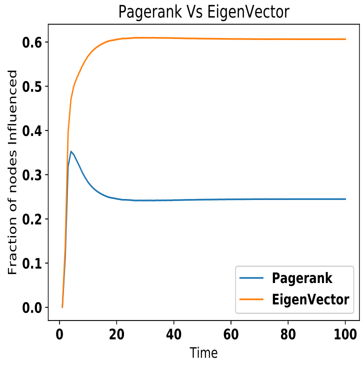


(a.) Pagerank vs EigenVector

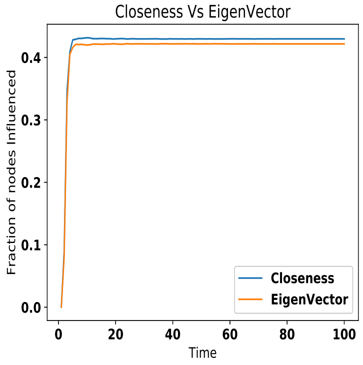


(b.) Closeness vs EigenVector

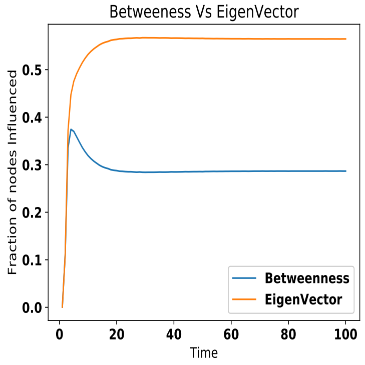


(c.) Betweenness vs EigenVector

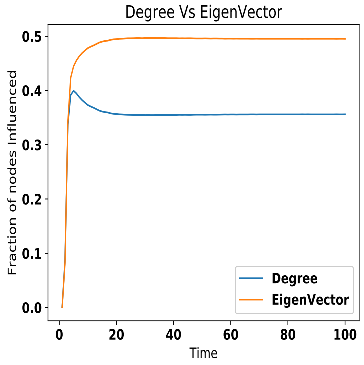


(d.) Degree vs EigenVector

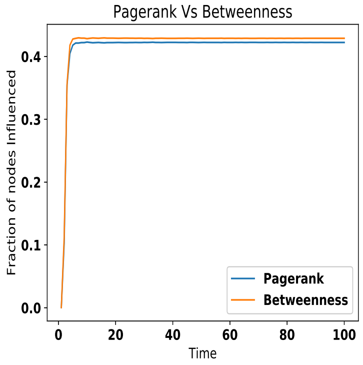


(e.) Pagerank vs Betweenness

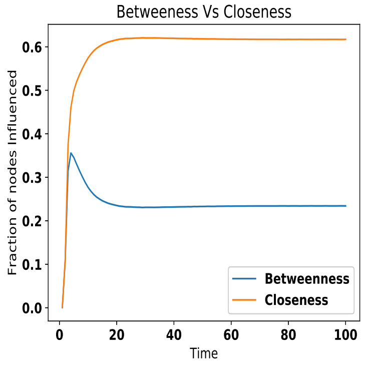


(f.) Betweenness vs Closeness

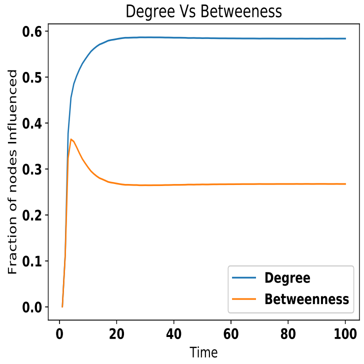


(g.) Degree vs Betweenness

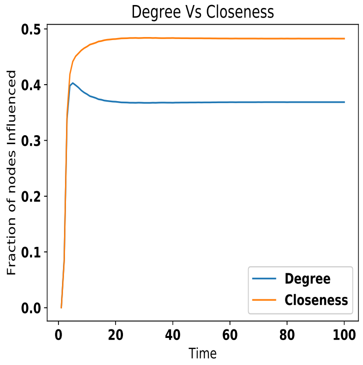


(h.) Degree vs Closeness

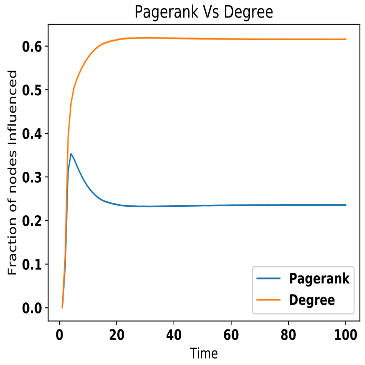


(i.) Pagerank Vs Degree

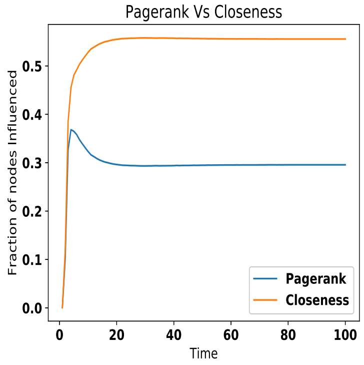


(j.) Pagerank Vs Closeness

Figure S1: Competition of various Centrality measures with each other for Wiki Vote Network

## 2 SUPPLEMENTARY DATA FOR CHESS COLLABORATION NETWORK

**Table S2.** This set of tables show the seed nodes of each centrality methods for Chess interaction network. Seed nodes here are the unique nodes selected from the top 10 nodes of each centrality measures. Blue coloured cells show the nodes which are not unique and can't be considered as a seed node if the distribution of seed nodes is unbiased.

| | | | | | | | | | | |
|---|---|---|---|---|---|---|---|---|---|---|
| **Pagerank** | 92 | 66 | 330 | 461 | 1623 | 237 | 1201 | 455 | 112 | 456 |
| **EigenVector** | 455 | 456 | 330 | 659 | 467 | 461 | 98 | 731 | 92 | 623 |

| | | | | | | | | | | |
|---|---|---|---|---|---|---|---|---|---|---|
| **Closeness** | 330 | 92 | 455 | 66 | 1201 | 456 | 237 | 461 | 659 | 573 |
| **EigenVector** | 455 | 456 | 330 | 659 | 467 | 461 | 98 | 731 | 92 | 623 |

| | | | | | | | | | | |
|---|---|---|---|---|---|---|---|---|---|---|
| **Betweenness** | 66 | 112 | 1623 | 1201 | 720 | 92 | 778 | 42 | 387 | 595 |
| **EigenVector** | 455 | 456 | 330 | 659 | 467 | 461 | 98 | 731 | 92 | 623 |

| | | | | | | | | | | |
|---|---|---|---|---|---|---|---|---|---|---|
| **Degree** | 330 | 92 | 461 | 455 | 66 | 456 | 467 | 237 | 659 | 1201 |
| **EigenVector** | 455 | 456 | 330 | 659 | 467 | 461 | 98 | 731 | 92 | 623 |

| | | | | | | | | | | |
|---|---|---|---|---|---|---|---|---|---|---|
| **Pagerank** | 92 | 66 | 330 | 461 | 1623 | 237 | 1201 | 455 | 112 | 456 |
| **Betweenness** | 66 | 112 | 1623 | 1201 | 720 | 92 | 778 | 42 | 387 | 595 |

| | | | | | | | | | | |
|---|---|---|---|---|---|---|---|---|---|---|
| **Betweenness** | 66 | 112 | 1623 | 1201 | 720 | 92 | 778 | 42 | 387 | 595 |
| **Closeness** | 330 | 92 | 455 | 66 | 1201 | 456 | 237 | 461 | 659 | 573 |

| | | | | | | | | | | |
|---|---|---|---|---|---|---|---|---|---|---|
| **Degree** | 330 | 92 | 461 | 455 | 66 | 456 | 467 | 237 | 659 | 1201 |
| **Betweenness** | 66 | 112 | 1623 | 1201 | 720 | 92 | 778 | 42 | 387 | 595 |

| | | | | | | | | | | |
|---|---|---|---|---|---|---|---|---|---|---|
| **Degree** | 330 | 92 | 461 | 455 | 66 | 456 | 467 | 237 | 659 | 1201 |
| **Closeness** | 330 | 92 | 455 | 66 | 1201 | 456 | 237 | 461 | 659 | 573 |

| | | | | | | | | | | |
|---|---|---|---|---|---|---|---|---|---|---|
| **Pagerank** | 92 | 66 | 330 | 461 | 1623 | 237 | 1201 | 455 | 112 | 456 |
| **Degree** | 330 | 92 | 461 | 455 | 66 | 456 | 467 | 237 | 659 | 1201 |

| | | | | | | | | | | |
|---|---|---|---|---|---|---|---|---|---|---|
| **Pagerank** | 92 | 66 | 330 | 461 | 1623 | 237 | 1201 | 455 | 112 | 456 |
| **Closeness** | 330 | 92 | 455 | 66 | 1201 | 456 | 237 | 461 | 659 | 573 |

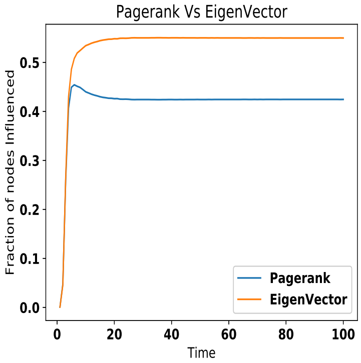


(a.) Pagerank vs EigenVector

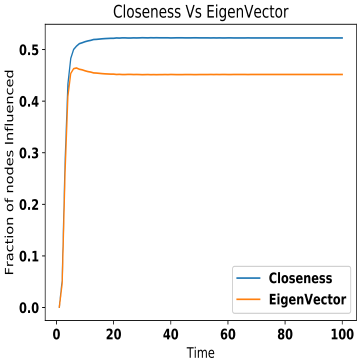


(b.) Closeness vs EigenVector

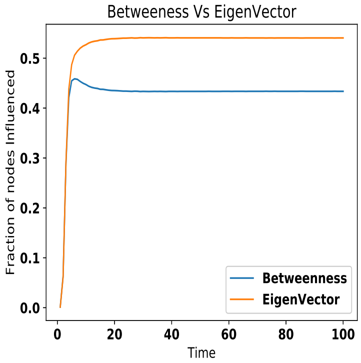


(c.) Betweenness vs EigenVector

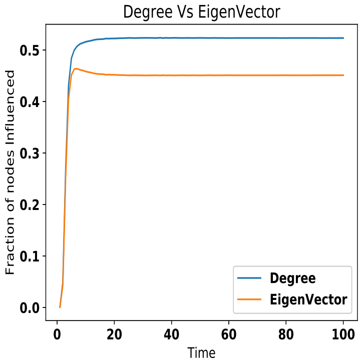


(d.) Degree vs EigenVector

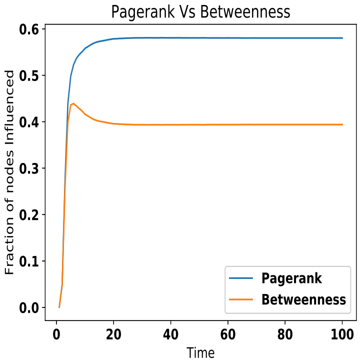


(e.) Pagerank vs Betweenness

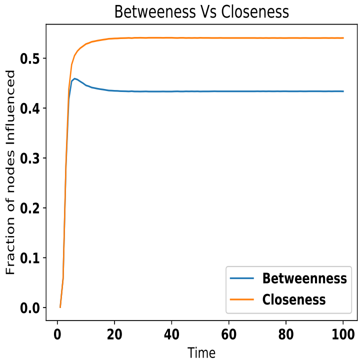


(f.) Betweenness vs Closeness

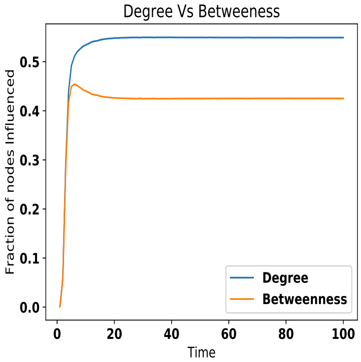


(g.) Degree vs Betweenness

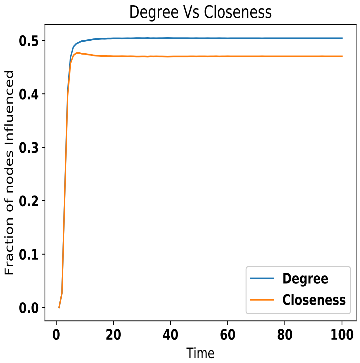


(h.) Degree vs Closeness

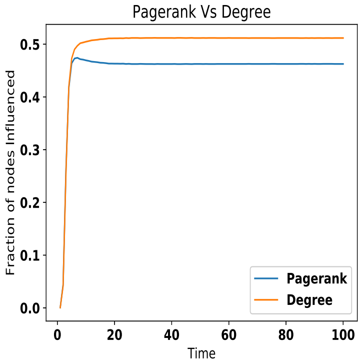


(i.) Pagerank Vs Degree

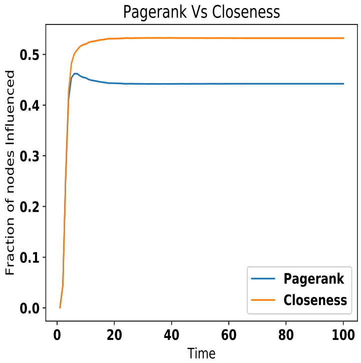


(j.) Pagerank Vs Closeness

Figure S2: Competition of various Centrality measures with each other for Chess Interaction Network

# 3 SUPPLEMENTARY DATA FOR HUMAN INTERACTION NETWORK

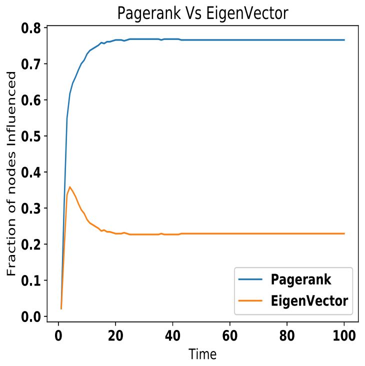


(a.) Pagerank vs EigenVector

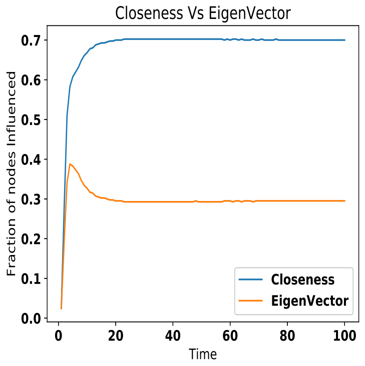


(b.) Closeness vs EigenVector

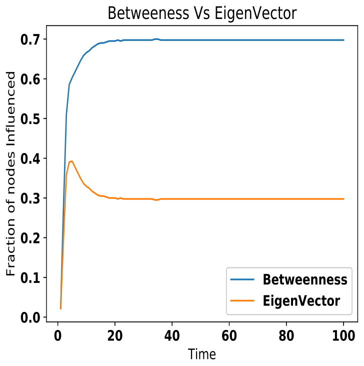


(c.) Betweenness vs EigenVector

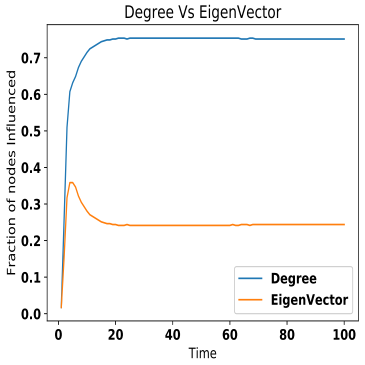


(d.) Degree vs EigenVector

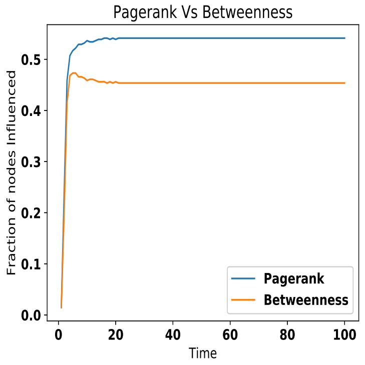


(e.) Pagerank vs Betweenness

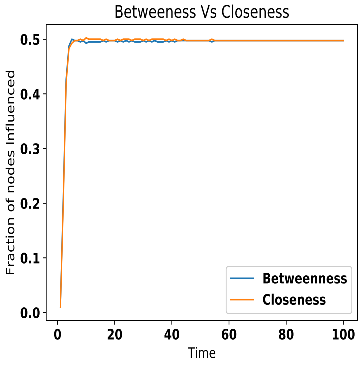


(f.) Betweenness vs Closeness

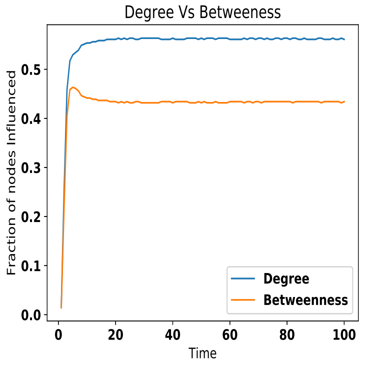


(g.) Degree vs Betweenness

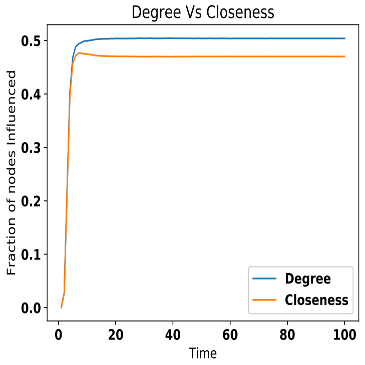


(h.) Degree vs Closeness

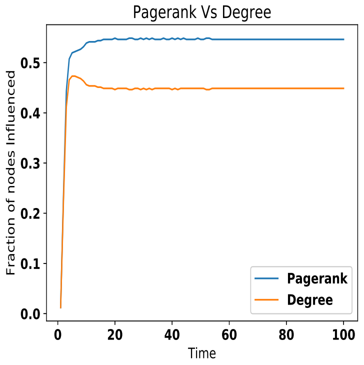


(i.) Pagerank Vs Degree

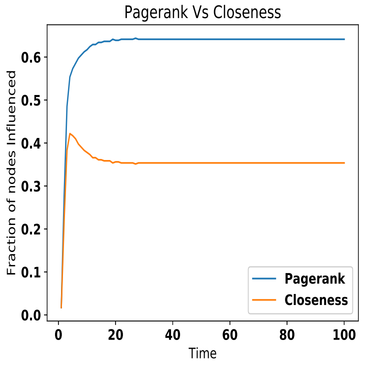


(j.) Pagerank Vs Closeness

Figure S3: Competition of various Centrality measures with each other for Human Interaction Network
